# New Synthesis and Antiparasitic Activity of Model 5-Aryl-1-methyl-4-nitroimidazoles

**DOI:** 10.3390/molecules14082758

**Published:** 2009-07-27

**Authors:** Haythem A. Saadeh, Ibrahim M. Mosleh, Mustafa M. El-Abadelah

**Affiliations:** 1 Chemistry Department, Faculty of Science, University of Jordan, Amman 11942, Jordan; 2 Department of Biological Sciences, Faculty of Science, University of Jordan, Amman 11942, Jordan

**Keywords:** 5-chloro-1-methyl-4-nitroimidazole, arylboronic acids, Suzuki coupling, 5-aryl-1-methyl-4-nitroimidazoles, antiparasitic activity

## Abstract

A number of 5-aryl-1-methyl-4-nitroimidazoles **5a-f** have been synthesized in good yields by the Suzuki coupling reaction between 5-chloro-1-methyl-4-nitroimidazole (**3**) and arylboronic acids **4a-f**, aided by dichlorobis-(triphenylphosphine)palladium(II), K_2_CO_3,_ and tetrabutylammonium bromide in water at 70-80 °C. Compounds **5a-f** were characterized by elemental analysis, NMR and MS spectral data. On the basis of *in vitro* screening data, 5-(3-chlorophenyl)-1-methyl-4-nitro-1*H*-imidazole (**5f**) exhibited potent lethal activity against *Entamoeba histolytica* and *Giardia intestinalis* with IC_50_ = 1.47 µM/mL, a value lower by a factor of two than that of the standard drug, metronidazole. The boosted activity of **5f** was not accompanied by any increased cytotoxicity. The rest of the series also exhibited potent antiparasitic activity with IC_50_ values in the 1.72-4.43 µM/mL range. The cytotoxicity of the derivatives **5c** and **5e** was increased compared to the precursor compound, metronidazole, although they remain non-cytotoxic at concentrations much higher than the antiparasitic concentration of the two derivatives.

## Introduction

**Figure 1:**
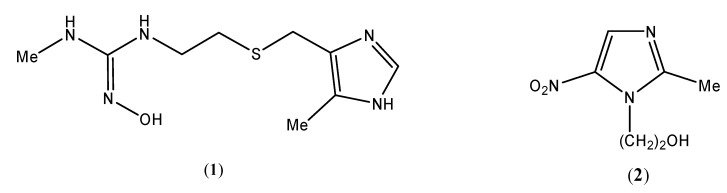
Model imidazole-based synthetic drugs.

The imidazole nucleus occurs naturally in *L*-histidine, histamine (a vasodelator hormone), thiamine (vitamin B_1_), and in several other biomolecules [1,2,3,4]. An example of an imidazole-containing synthetic drugs is cimetidine (**1**, Figure 1)**,** widely used for the treatment of peptic ulcers [5,6,7,8]. Nitroimidazoles have wide applications in the drug synthesis due to their biological activity. Examples include natural azomycin (antibiotic, 2-nitroimidazole) [9,10,11,12] and synthetic metronidazole (**2,** anti-amoebic dysentry) [13,14,15,16] (Figure 1).

(Substituted)-4-nitroimidazoles also showed activity against various microbes and found applications in chemotherapy [17,18,19]. Thus, a series of 1-methyl-4-nitro-5-substituted imidazoles were reported to exhibit antileishmanial, antiamebic and anthelmintic activities [17]. Also, a number of synthetic 5-(substituted azolyl)-1-methyl-4-nitroimidazoles were reported to exhibit anti-parasitic activity [20,21,22].

**Scheme 1:**
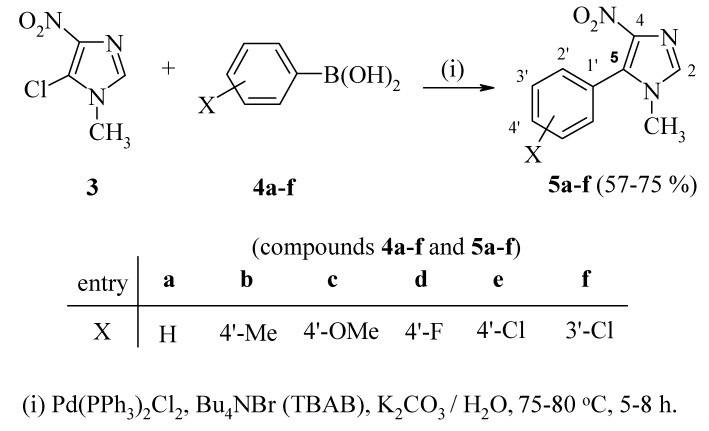
Synthesis of 5-aryl-1-methyl-4-nitroimidazoles **5a-f** *via* Suzuki coupling.

Herein, we wish to report a one-pot synthesis of 5-aryl-1-methyl-4-nitroimidazoles **5a-f** by reaction of 5-chloro-1-methyl-4-nitroimidazole (**3**) with arylboronic acids **4** under Suzuki reaction conditions [25,26,27,28,29,30] (Scheme 1), and the evaluation of their antiparasitic activity.

## Results and Discussion

### Chemistry

**Scheme 2:**
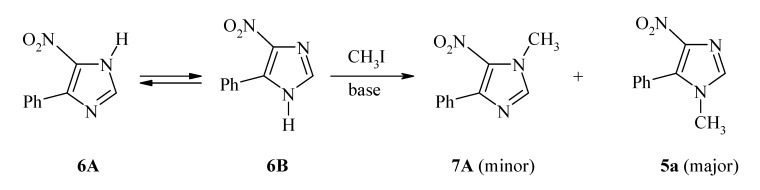
*N*-Methylation of 5(4)-aryl-4(5)-nitroimidazole [23].

Previously, the synthesis of 5-aryl-1-methyl-4-nitroimidazoles (exemplified by **5a/**Scheme 2) was achieved *via N*(1)-methylation of the respective 5(4)-aryl-4(5)-nitroimidazole (**6A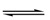 6B**) [23]. This step led to a mixture of **5a** and its isomeric 1-methyl-4-aryl-5-nitroimidazole **7A**, the isolation of which required extra separation and purification efforts. Additionally, the required synthon (**6A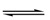 6B**) was prepared by condensation of (substituted)phenacyl chloride with formamide, followed by acidification and then neutralization with aqueous ammonium hydroxide [24]. 

In the present work, reaction of the commercially available 5-chloro-1-methyl-4-nitroimidazole (**3**) with arylboronic acids **4** under Suzuki reaction conditions provided an efficient route towards the formation of 5-aryl-1-methyl-4-nitroimidazoles **5a-f**, as outlined in Scheme 1.

Compared to the multistep synthetic route noted above, the direct Suzuki coupling reaction provides a more convenient and efficient route for the preparation of 5-aryl-1-methyl-4-nitroimidazoles **5a-f**. Thus, treatment of 5-chloro-1-methyl-4-nitroimidazole (**3**) with arylboronic acids **4a-f** using 3 mole % of dichloro bis-(triphenylphosphin)palladium(II), K_2_CO_3,_ and tetrabutylammonium bromide in water at 70-80 °C, gave the corresponding 5-arylimidazole derivatives **5a-f** [31]. These Pd-catalyzed cross-coupling reactions proceeded nicely in the presence of the ‘additive’ (TBAB). The latter is known to stabilize colloidal palladium nanoparticles that act as catalysts in the Suzuki coupling of aryl halides [32], and thus enhances the rate of the coupling reaction. The presence of the strongly electron-withdrawing nitro group on the imidazole ring facilitates the coupling process which results in good yields of **5a-f**.

The microanalytical and spectral (HRMS and NMR) data, given in the Experimental part, are in accordance with the assigned structures for compounds **5a-f**. Thus, the observed high resolution MS data for M^+^ are in good agreement with the values calculated for the respective molecular formulas. The NMR spectra displayed ^1^H- and ^13^C- signals characteristic of the respective aryl moieties introduced at the C-5. Assignments of the ^1^H- and ^13^C- signals to the different respective protons and carbons are based on DEPT and 2D (COSY, HMQC, HMBC) experiments which showed correlations consistent with these assignments. The ^1^H-NMR spectra for compounds **5a-f** show aromatic protons for the aryl group introduced by Suzuki coupling besides the imidazole C-2 proton at ~ 7.45 ppm. The CH_3_ and OCH_3_ aryl substituents for compounds **5b** and **5c** appear at 2.41 and 3.85 ppm, respectively. Compound **5d** containing 4'- fluorine substituent showed additional coupling caused by the ^19^F nucleus with a vicinal coupling ^1^H-^19^F (^3^*J*= 8.5 Hz). The ^13^C-NMR spectra of the prepared compounds **5a-f** showed carbon signals expected for aryl groups introduced by Suzuki coupling besides the imidazole C-2, C-4 and C-5 carbons. In compound **5d**, the skeletal carbons of the benzo ring at C-5 are readily recognizable by their signal splitting 'as doublets' arising from spin-spin coupling with the nearby fluorine atom at C-4' with varying *J*_C-F_ values for the four different carbons C1'-C4' (^1^*J* = 250 Hz ; ^2^*J* = 22 Hz; ^3^*J* = 8.6 Hz ; ^4^*J* = 3.6 Hz). DEPT experiments were employed to differentiate primary and tertiary carbons from quaternary carbons.

### Antiamoebic and antigiardial activity

The antiamoebic and antigiardial activities of the compounds **5a-f** were investigated using *in vitro* bioassays that included the standard antiamoebic and antigiardial drug metronidazole as a control. The cytotoxicity of the compounds on the two cell lines, Hep-2 and Vero cells, was also compared with that of metronidazole. The IC_50_ values of the compounds against *Entamoeba histolytica*, *Giardia intestinalis*, and the two cell lines are given in Table 1. As indicated in the Table, all the tested compounds showed biological activities against *Entamoeba* and *Giardia*. Compounds **5c**, **5e**, and **5f** showed the highest activity against the parasites, with IC_50_ values ranging from around one to two micromolar, compared to around four micromolar for the standard drug, metronidazole. When the cytotoxicity of the prepared molecules is considered, compound **5f** appears to be the best among the prepared derivatives of metronidazole, as indicated in Table 2. This Table presents the IC_50_ ratio of metronidazole over that of the derivative compounds **5a-f** against the parasites and the two investigated cell lines. Compound **5f** was around two to three times more active than metronidazole, against *Entamoeba* and *Giardia*, respectively. Fortunately, the boosted activity of this compound was not accompanied with any increased cytotoxicity. In contrast, the cytotoxicity of the derivatives **5c** and **5e** has increased compared to the precursor compound, metronidazole, although they remain non-cytotoxic at concentrations much higher than the antiparasitic concentration of the two derivatives. As can be concluded from Table 1, the IC_50_ values of compounds **5c** and **5e** against the two cell lines were ≥ 230 times higher than that against the parasites under investigation.

Interestingly, the tested compounds exhibited an almost similar pattern of activity against both *G. intestinalis* and *E. histolytica* (Table 1), indicating that each compound affects both parasites by a similar mechanism of action. In addition, the molecular modifications on our derivatives did not render any of the compounds inactive. The other molecules, **5a**, **5b**, and **5d** remained as active as metronidazole (Table 1,Table 2).

The activities exhibited by the derivatives, especially compound **5f**, suggest that the derivatives may be used as new lead compounds in the development of new antiparasitic drugs. Although drug resistance to *Entamoeba* and *Giardia* does not, so far, appear to be a serious problem, occasional reports of failure with metronidazole [33] and the reported variations in drug sensitivities of isolates [34] may be alarming. Therefore, the importance of such biologically active, non-cytotoxic metronidazole derivatives, especially **5f**, lies in their potential contribution to overcome the problem of resistance of pathogens to the standard drugs. Additionally, because of the limited number of drugs available in the market against anaerobic protozoal parasites and bacteria there is a serious need for new active compounds. The molecular modification on the original drugs, therefore, offers alternatives that may bypass the already developed mechanisms adopted by the anaerobic pathogens against the standard drugs. Our derivatized compounds (**5c**, **5e**, **5f**) are good drug candidates to be tested against metronidazole-resistant parasites and bacteria.

**Table 1:** Antiparasitic activities of compounds **5a-f**.

**Mean IC50 ± SD^(*n*)^ (μM)**				
**Compound**	*Giardia intestinalis*	*Entamoeba histolytica*	Hep-2 cells	Vero cells
**5a**	4.43 ± 1.97	4.04 ± 0.28	1040.27 ± 19.18	1748.28 ± 18.38
**5b**	4.01 ± 0.75	3.10 ± 0.41	1610.74 ± 22.23	1633.32 ± 13.61
**5c**	1.72 ± 0.57	1.16 ± 0.19	568.80 ± 22.71	868.24 ± 22.02
**5d**	3.76 ± 0.2	4.39 ± 0.71	1894.12 ± 21.13	1918.41 ± 13.37
**5e**	1.90 ± 0.14	1.56 ± 0.156	437.19 ± 16.39	725.05 ± 11.79
**5f**	1.47 ± 0.14	1.89 ± 0.14	1780.21 ± 15.71	1783.16 ± 19.66
Metronidazole	4.39 ± 0.59	4.10 ± 0.78	2044.20 ± 26.36	2071.35 ± 16.37

SD^(*n*)^: Standard deviation

**Table 2:** IC_50_ ratios.

**IC_50_ Ratio**				
**(metronidazole/compound)**				
**Compound**	*Giardia intestinalis*	*Entamoeba histolytica*	Hep-2 cells	Vero cells
**5a **	1	1	2	1.2
**5b**	1.1	1.3	1.3	1.3
**5c**	3.5	2.6	3.6	2.4
**5d **	1.2	0.93	1.1	1.1
**5e**	2.6	2.3	4.7	2.9
**5f**	2.2	2.9	1.2	1.2

## Experimental

### General

The following chemicals, employed in this study, were purchased and used without further purification: 5-chloro-1-methyl-4-nitroimidazole, phenylboronic acid, 4-tolylboronic acid, 4-methoxy-phenylboronic acid, 4-flourophenylboronic acid, 4-chlorophenylboronic acid, 3-chlorophenylboronic acid, tetrabutyl ammonium bromide and dichloro bis-(triphenylphosphin)palladium(II). Melting points (uncorrected) were determined on a Gallenkamp electrothermal melting temperature apparatus. ^1^H- and ^13^C-NMR spectra were recorded on a Bruker DPX-300 instrument with TMS as internal reference. High-resolution mass spectra (HRMS) were measured in positive ion mode using electrospray ion trap (ESI) technique by collision-induced dissociation on a Bruker APEX-4 (7 Tesla) instrument. The samples were dissolved in acetonitrile, diluted in spray solution (methanol/water 1:1 v/v + 0.1% formic acid) and infused using a syringe pump with a flow rate of 2 µL/min. External calibration was conducted using Arginine cluster in a mass range *m/z* 175-871. IR spectra were recorded as KBr discs on a Nicolet Impact-400 FT-IR spectrophotometer. Elemental analyses were preformed at the Microanalytical Laboratory of the Hashemite University, Zarqa-Jordan, and the results were found to be in good agreement (± 0.4%) with the calculated values.

### General procedure for the synthesis of 5-aryl-1-methyl-4-nitroimidazoles ***5a-f***

A mixture of 5-chloro-1-methyl-4-nitroimidazole **3 (**4 mmol), the particular arylboronic acid **4a-f** (4 mmol), Pd(PPh_3_)_2_Cl_2_ (3 mol%, 80 mg), powdered K_2_CO_3_ (1.4 g, 10 mmol) and Bu_4_NBr (1.3 g, 4 mmol) in water (3 mL) was heated with stirring at 75-80 ºC for 5-8 h. Thereafter, the reaction mixture was cooled, poured into water (25 mL) and extracted with dichloromethane (2 x 30 mL). The combined organic extracts were dried over anhydrous sodium sulfate and the solvent was removed under reduced pressure. The residue was purified by column chromatography with chloroform-methanol (95:5 v/v) to afford the respective compounds **5a-f**.

*1-Methyl-4-nitro-5-phenyl-1H-imidazole* (**5a**): Yield 0.73 g (70%); mp 177-179 °C (Lit. [23] 178-180 ^o^C). ^1^H-NMR (CDCl_3_): *δ* 3.51 (s, 3H, *N*-CH_3_), 7.36 (m, 2H, 2'-H + 6'-H ), 7.46 (s, 1H, 2-H), 7.50 (m, 3H, 3'-H + 4'-H+ 5'-H ); ^13^C-NMR (CDCl_3_): *δ* 33.1 (CH_3_), 126.5 (C), 128.9 (C-2'+ C-6'), 130.1 (C-3'+ C-5'), 130.2 (C-4'), 132.6 (C-5), 135.6 (C-2), 140.1 (C-4); HRMS ((+ve)-ESI): *m/z* calcd. for C_10_H_9_N_3_O_2_Na [M+Na]^+^: 226.05870, found: 226.05870; Anal. Calcd**.** for C_10_H_9_N_3_O_2_ (203.20): C, 59.11; H, 4.46; N, 20.68. Found: C, 59.50; H, 4.77; N, 20.54.

*1-Methyl-5-(4-methylphenyl)-4-nitro-1H-imidazole* (**5b**)**:** Yield 0.57 g (65%); mp 179-181 °C. ^1^H-NMR (CDCl_3_): *δ* 2.41 (s, 3H, 4'-CH_3_), 3.50 (s, 3H, *N*-CH_3_), 7.25 (d, *J* = 8 Hz, 2H, 2'-H+ 6'-H), 7.31 (d, *J* = 8 Hz, 2H, 3'-H+ 5'-H), 7.45 (s, 1H, 2-H); ^13^C-NMR (CDCl_3_): *δ* 21.6 (4'-CH_3_), 33.1 (*N*-CH_3_), 115.0 (C-1'), 123.4 (C-4'), 129.6 (C-3'+ C-5'), 129.9 (C-2'+ C-6'), 130.6 (C-5), 135.6 (C-2), 140.5 (C-4); HRMS ((+ve)-ESI): *m/z* calcd. for C_11_H_11_N_3_O_2_Na [M+Na]^+^: 240.07435, found: 240.07435; Anal. Calcd**.** for C_11_H_11_N_3_O_2_ (217.22): C, 60.82; H, 5.01; N, 19.34. Found: C, 61.03; H, 5.15; N, 19.05.

*5-(4-Methoxyphenyl)-1-methyl-4-nitro-1H-imidazole* (**5c**)**:** Yield 0.70 g (75%); mp 182-184 °C. ^1^H-NMR (CDCl_3_): *δ* 3.51 (s, 3H, *N*-CH_3_), 3.86 (s, 3H, *O*-CH_3_), 7.01 (d, *J* = 8.8 Hz, 2H, 3'-H+ 5'-H ), 7.31 (d, *J* = 8.8 Hz, 2H, 2'-H+ 6'-H), 7.45 (s, 1H, 2-H); ^13^C-NMR (CDCl_3_): *δ* 33.1 (*N*-CH_3_), 55.5 (*O*-CH_3_), 114.4 (C-3'+ C-5'), 118.2 (C-1'), 131.6 (C-2'+ C-6'), 132.6 (C-5), 135.4 (C-2), 141.0 (C-4), 160.9 (C-4'); ); HRMS ((+ve)-ESI): *m/z* calcd. for C_11_H_12_N_3_O_3_ [M+H]^+^: 234.08732, found: 234.08732; Anal. Calcd**.** for C_11_H_11_N_3_O_3_ (233.22): C, 56.65; H, 4.75; N, 18.02. Found: C, 56.72; H, 4.82; N, 17.78.

*5-(4-Fluorophenyl)-1-methyl-4-nitro-1H-imidazole* (**5d**)**:** Yield 0.65 g (73%); mp 137-139 °C. ^1^H-NMR (CDCl_3_): *δ* 3.52 (s, 3H, *N*-CH_3_), 7.20 (m, 2H, 2'-H + 6'-H), 7.38 (m, 2H, 3'-H + 5'-H), 7.47 (s, 1H, H-2); ^13^C-NMR (CDCl_3_): *δ* 33.1 (CH_3_), 116.3 (d, ^2^*J*_C-F_ = 22 Hz, C-3'+ C-5'), 122.4 (d, ^4^*J*_C-F_ = 3.6 Hz, C-1'), 131.0 (C-5), 132.3 (d, ^3^*J*_C-F_ = 8.6 Hz, C-2'+ C-6'), 135.6 (C-2), 141.0 (C-4), 163.7(d, ^1^*J*_C-F_ = 250 Hz, C-4'); HRMS ((+ve)-ESI): *m/z* calcd. for C_10_H_8_FN_3_O_2_Na [M+Na]^+^: 244.04928, found: 244.04928; Anal. Calcd**.** for C_10_H_8_FN_3_O_2_ (221.19): C, 54.30; H, 3.65; N, 19.00. Found: C, 53.98; H, 3.49; N, 18.80.

*5-(4-Chlorophenyl)-1-methyl-4-nitro-1H-imidazole* (**5e**)**:** Yield 0.54 g (57%); mp 237-239 °C (Lit. [24] 238-240 ^°C^).^1^H-NMR (CDCl_3_): *δ* 3.52 (s, 3H, *N*-CH_3_), 7.32 (d, *J* = 8.6 Hz, 2H, 2'-H + 6'-H), 7.48 (s, 1H, 2-H), 7.49 (d, *J* = 8.6 Hz, 2H, 3'-H + 5'-H); ^13^C-NMR (CDCl_3_): *δ* 33.2 (CH_3_), 116.6 (C-1'), 124.9 (C-4'), 129.3 (C-2'+ C-6'), 130.2 (C-5), 131.5 (C-3'+ C-5'), 135.8 (C-2), 141.1 (C-4); HRMS ((+ve)-ESI): *m/z* calcd. for C_10_H_9_ClN_3_O_2_ [M+H]^+^: 238.03778, found: 238.03778; Anal. Calcd**.** for C_10_H_8_ClN_3_O_2_ (237.64): C, 50.54; H, 3.39; N, 17.68. Found: C, 50.33; H, 3.28; N, 17. 32.

*5-(3-Chlorophenyl)-1-methyl-4-nitro-1H-imidazole* (**5f**)**:** Yield 0.60 g (63%); mp 170-172 °C. ^1^H-NMR (CDCl_3_): *δ* 3.50 (s, 3H, *N*-CH_3_), 7.26 (m, 1H), 7.36 (m, 1H), 7.45 (s, 1H, 2-H), 7.48 (m, 2H); ^13^C-NMR (CDCl_3_): *δ* 33.2 (CH_3_), 120.3 (C-1'), 128.4 (C-6'), 130.1 (C-2'), 130.3 (C-4'), 130.5 (C-5'), 130.9 (C-5), 134.9 (C-3'), 135.8 (C-2), 141.4 (C-4); HRMS ((+ve)-ESI): *m/z* calcd. for C_10_H_9_ClN_3_O_2_ [M+H]^+^: 238.03778, found: 238.03778; Anal. Calcd**.** for C_10_H_8_ClN_3_O_2_ (237.64): C, 50.50; H, 3.39; N, 17.68. Found: C, 50.90; H, 3.18; N, 17.75.

### Biological Activity

#### Test organisms

*Entamoeba histolytica* HK-9 strain (ATCC number 30015) was cultured in LYI-S-2 medium supplemented with antibiotics. *Giardia intestinalis* WB strain (ATCC number 30957) was grown in a modified YI-S medium with antibiotics. Both parasites were grown as described [35]. Briefly, the parasites were cultivated and maintained in 15-mL screw-capped borosilicate glass tubes. *Entamoeba* and *Giardia* were harvested from confluent cultures by chilling of the tubes on ice, followed by centrifugation. 

#### Antiamoebic and antigiardial activity

The antiamoebic and antigiardial activities of the prepared molecules and metronidazole as the standard antiamoebic and antigiardial drug were tested as described [36]. Briefly, the tested compounds and metronidazole were dissolved in dimethyl sulfoxide (DMSO) then in medium and filter-sterilized. Two-fold dilutions starting at 15 μg/mL were prepared in a final volume of 15 mL to exclude air from the tube. Each tube was inoculated with 20,000 cells of the parasite under testing (*Entamoeba* or *Giardia*). Each compound was assayed in duplicate in each of three independent experiments. In each assay, the appropriate controls were performed, including the one without any compound and another with metronidazole as the positive control. The biological activity of the compounds was evaluated by counting the parasites in each tube using the standard hemacytometer. In each count, trypan blue was employed to distinguish live from dead parasites [37].

#### Cytotoxicity assay

The cytotoxicity of the reported compounds and the reference drug, metronidazole, was investigated on Hep-2 and Vero cells using the standard cytotoxicity assay and the trypan blue exclusion method as described before [36]. Briefly, 100 μL portions of each cell suspension were added to the wells of 96-well plates, incubated for 24 h, and the medium in each well was then replaced with 150 μL fresh medium. Solutions of the compounds or the reference drug were dissolved in DMSO, prepared in medium, and filter sterilized. Then, 150 μL-two fold serial dilutions of each of the compounds and the reference drug starting at a concentration of 2,000 μg/mL in culture medium were prepared in the plates. After 48 hour incubation, the number of cells in each well was determined a hemacytometer. Each compound was assayed in duplicate in each of three independent experiments. In each assay the negative controls (without any compound or reference drug) were included in duplicates.

## Conclusions

In conclusion, we have described the synthesis of a number of 5-aryl-1-methyl-4-nitroimidazoles **5a-f** with promising antiparasitic activity. Bioassay of these compounds indicated significant antiparasitic activities against *Entamoeba histolytica* and *Giardia intestinalis* that they could be used as lead structures for the development of antiparasitic drugs. The IC_50_ of the hybrid molecule **5f** was found to be about three times lower than that of the standard drug metronidazole against those parasites and it could therefore be considered as a good drug candidate to be tested against metronidazole-resistant parasites and possibly anaerobic bacteria.
